# Effect of a Polyphenol-Based Additive in Pig Diets in the Early Stages of Growth

**DOI:** 10.3390/ani11113241

**Published:** 2021-11-12

**Authors:** Gianluca Galassi, Marco Battelli, Nicole Verdile, Luca Rapetti, Raffaella Zanchi, Sharon Arcuri, Francesca Petrera, Fabio Abeni, Gianni Matteo Crovetto

**Affiliations:** 1 Dipartimento di Scienze Agrarie e Ambientali—Produzione, Territorio, Agroenergia, Università degli Studi di Milano, Via Celoria 2, 20133 Milano, Italy; gianluca.galassi@unimi.it (G.G.); nicole.verdile@unimi.it (N.V.); luca.rapetti@unimi.it (L.R.); matteo.crovetto@unimi.it (G.M.C.); 2 Dipartimento di Scienze per gli Alimenti, la Nutrizione e l’Ambiente, Università degli Studi di Milano, Via Celoria 2, 20133 Milano, Italy; raffaella.zanchi@unimi.it; 3 Dipartimento di Scienze Veterinarie per la Salute, la Produzione Animale e la Sicurezza Alimentare, Via dell’Università 6, 26900 Lodi, Italy; sharon.arcuri@unimi.it; 4 Centro di Ricerca Zootecnia e Acquacoltura, Consiglio per la Ricerca in Agricoltura e l’Analisi dell’Economia Agraria, Via Antonio Lombardo 11, 26900 Lodi, Italy; francesca.petrera@crea.gov.it (F.P.); fabiopalmiro.abeni@crea.gov.it (F.A.)

**Keywords:** weaned piglets, feed additives, gastrointestinal tract health, piglet gut health, pig intestinal microbiota, digestibility

## Abstract

**Simple Summary:**

The health of piglets, especially the health of the gastrointestinal tract, is subject to severe stress during weaning. The use of antibiotics can help piglets to counteract the negative effects of this period. However, the use of antibiotics as growth promoters can increase the risk of antibiotic resistance. For this reason, antibiotic replacement products are sought to promote gut health. We tested the commercial product GreenFIS^®^ (New Feed Team Ltd., Lodi, Italy), a blend of natural substances acknowledged as beneficial for gut health, at two levels of inclusion, compared to a control diet, on 60 piglets. No differences were recorded between diets regarding the growth performance. The gut histological analysis performed on some animals did not reveal any difference between diets. Microbiological analyses were also performed on the feces and the piglets fed GreenFIS^®^ showed a higher number of a group of bacteria positively associated with starch utilization. After the post-weaning trial, a digestibility study was conducted on six pigs randomly chosen for each treatment. The addition of GreenFIS^®^ to the diet led to higher digestibility compared to the control, although it did not reveal, in the excellent hygienic conditions of the experimental farm, a positive effect on the gut heath.

**Abstract:**

The weaning period is a stressful period for the gastrointestinal tract (GIT) of piglets. This work aims to evaluate the effects of the commercial polyphenol-based product GreenFIS^®^ on: (1) GIT health and performance of 60 weaned piglets; (2) digestibility in 18 growing pigs. Three diets were tested: a control diet (C), C plus 2.5 g of GreenFIS^®^/kg C (T1), and C plus 5 g of GreenFIS^®^/kg C (T2). After the post-weaning trial three piglets per treatment were sacrificed for the GIT histological analysis. No differences between diets were recorded in terms of growing performance or clinical and biochemical blood parameters. The GIT histological analysis did not show any indicators of inflammation for any of the groups. The feces of the two extreme treatments (C and T2) were analyzed for microbiota, revealing a greater presence of the *Ruminococcus bromii* group, positively associated with starch degradation, in T2. In the second experiment six pigs per treatment were randomly chosen for the digestibility study. The inclusion of GreenFIS^®^ at both levels led to a higher fecal digestibility of gross energy (86.2%, 89.1%, and 89.5%, for C, T1, and T2, respectively) and crude protein (87.0%, 90.2%, and 90.0%). In conclusion, the additive did not improve, in the excellent experimental hygienic conditions, the gut health, but it did increase nutrient digestibility.

## 1. Introduction

The weaning period is a stressful and sensitive moment for piglets, during which the gastrointestinal tract (GIT) undergoes physiological changes [1]. Frequently, during the post-weaning period, low growth performance is common due to several factors (e.g., environmental, nutritional, and physiological) [2] and piglets are more susceptible to diarrhea [3]. Post-weaning diarrhea (PWD) is a multifactorial disease that arises, among other causes, from the loss of the passive protection provided by colostrum, the decreased nutrient intake and digestibility, increased oxidative stress, and changes in intestinal barrier integrity and function [3,4].

According to López-Gálvez et al. [5], effective digestion and absorption of feed, together with the animal’s status of wellbeing, represent some of the main criteria to evaluate the response to a dietary treatment of the piglet in terms of GIT health and function. Moreover, histological parameters such as villi atrophy, crypt hyperplasia and the villus/crypt ratio (V:C) represent a reliable tool to evaluate the effect of diet regimes and assess the general mucosa state health.

Antibiotics are used in animal husbandry for therapeutic use, for prophylactic use, and as growth promoters [6]. Due to the phenomenon of antibiotic resistance the European Union banned the use of antibiotics as growth promoters in January 2006 [1]. However, when antibiotics as growth promoters are banned there is often an increase in veterinary use of therapeutic antibiotics for weaning pigs [7,8]. Moreover, due to the worldwide increase in demand for foods of animal origin, the global consumption of antimicrobials will increase [9]. For these reasons, alternative management and feeding strategies are being sought in order to reduce the use of antibiotics for therapeutic purposes [7,10].

Among the additives available to compensate for the negative effects of removing antibiotic growth promoters, there are polyphenols, humic substances, short-chain fatty acids (SCFA), mannan-oligosaccharide (MOS), and yeasts.

Polyphenols are the most abundant bioactive compounds of plant extracts [10]. Polyphenols fed to pigs showed powerful antioxidant activity and the ability to alter intestinal microbiota, reducing the presence of potentially pathogenic bacteria [11,12,13,14].

Humic substances (HS) are organic residues originating from decaying organic matter of relatively high molecular weight that include humates, humifulvates, humic acids, and fulvic acid [15]. Humic acids have been used as antidiarrheal and immune-stimulatory agents in veterinary practice [16]. Moreover, humic acids could have a series of positive effects when fed to pigs, such as an increase in the average daily weight gain (ADG) and feed conversion ratio (FCR) [16,17].

SCFA are a group of molecules composed of one to seven carbon atoms mainly derived from intestinal bacterial fermentation of plant material. Of the SCFA, acetic, propionic, and butyric acids are the shortest and most abundant [18]. These three SCFA are commonly used as acidifiers in pig diets. Butyrate has been particularly studied for its supposed positive effects on growth performance when fed to gestating sows, piglets, and growing pigs [19,20,21]. Moreover, it is a promoter of gut tissue development and a regulator of the intestinal microbiota [18].

Mannan oligosaccharides (MOS) are non-digestible oligosaccharides commonly derived from the yeast cell wall [22]. Formally, MOS are classified as prebiotics; however, their mechanism of action differs from that of other prebiotics, as they are nutrients neither for the gastrointestinal microorganisms nor for the host [23]. For this reason, MOS are assigned to the nutricine group of substances capable of improving the animal’s health and increasing its performance [23]. MOS are useful in pig nutrition due to their several positive effects [24]. When fed to sows, MOS could increase colostrum production and quality, with positive effects on piglets’ growth performance [25]. In addition, MOS inhibits the binding of some pathogenic bacteria to the intestinal walls [26].

Yeast products make up a broad category of feed supplements, such as live yeast or yeast extracts, with possible positive effects on animals [27]. Nucleotide-rich yeast extract (NRYE) helps piglets to challenge weaning, stimulating the immune system and maintaining a healthy intestinal environment [28,29]. Moreover, NRYE enhances the ADG of weaned pigs and reduces the incidence of diarrhea [30,31].

The aim of this work was to evaluate the effects of the inclusion in the diet, at two different levels (2.5 and 5 g/kg) of the commercial product GreenFIS^®^ (New Feed Team Ltd, Lodi, Italy), based on polyphenols, humic acids, glycerides of butyric acid, MOS, and NRYE, on the development and health of the GIT and on the performance of weaned piglets. Subsequently, during the growing phase of the same pigs used in the previous trial, the digestibility and nitrogen balance of the two supplemented diets were evaluated.

## 2. Materials and Methods

The study was conducted at the Research Center “Cascina Baciocca” at Cornaredo (Milan) of the Università degli Studi di Milano (Italy). Animal procedures were conducted after obtaining authorization for the study (authorization no. 634/2017-PR) from the Ministry of Health and in accordance with the Italian law on animal welfare for experimental animals (Legislative Decree 26/2014).

The study involved two different sequential experiments that aimed at testing the effect of the additive GreenFIS^®^ at two different levels of inclusion in the diet (2.5 and 5 g/kg) on different parameters. The GreenFIS^®^ has the following percentage composition: 45% polyphenols from lignocellulose (namely hydrophilic polyphenols from hydrolyzed lignocellulosic materials), 13% humic acids, 7% fulvic acids, 19% glyceryl tributyrate (TRIB), 15% MOS, and 1% NRYE. The first trial was conducted on weaned piglets, while the second experiment was performed during the growing phase.

### 2.1. Experiment 1 (Post-Weaning Trial)

#### 2.1.1. Animals, Experimental Design, and Diets

Sixty 28 days old weaned piglets derived from PIC (Large White × Landrace) females and Goland C21 males, homogeneous for their body weight (BW), were used for the first experiment. Their initial BW was 9.0 ± 1.0 kg, on average. The piglets were randomly divided into 3 groups of 20 animals. Each group was randomly assigned to one of the three experimental diets: the control diet (C), the C diet plus 2.5 g of GreenFIS^®^ per kg of C (T1), and the C diet plus 5.0 g of GreenFIS^®^ per kg of C (T2). Five repetitions (pens) of four randomly selected piglets were then composed for each group. The four piglets of each repetition were reared in a post-weaning pen randomly located in a unique room. The stainless-steel pens with slatted floors had the following dimensions: 1.97 m × 1.07 m.

The ingredients and chemical composition of the C diet fed pigs during the two experiments are reported in Table 1. The diets were formulated to fulfill the National Research Council (NRC) requirements [32]. The inclusion of GreenFIS^®^ did not influence the main nutrient profiles of the diets.

All animals had ad libitum access to the assigned diet and to water.

All piglets were individually weighed at the beginning, in the middle, and at the end of the trial, which lasted 43 days, for the calculation of ADG. The total feed consumption of each pen was recorded daily for the calculation of FCR.

At the end of the experimental period, three pigs per treatment were sacrificed for the gut histological analysis.

#### 2.1.2. Clinical and Biochemical Parameters

Blood samples from 30 piglets (2 piglets per cage, 10 piglets per diet, randomly selected) were obtained at the end of the experiment by venipuncture from the jugular vein using two 5 mL evacuated tubes (one containing Li–heparin for biochemical profile and one without anticoagulant for serum protein profile), in the morning before feed distribution. Blood samples were cooled immediately by cooling bags and transported to the laboratory within 2 h after collection.

Li–heparin-treated tubes were centrifuged within 2 h after collection at 2850× *g* for 20 min at 4 °C; plasma was divided into aliquots and stored at −20 °C until biochemical metabolic profile analysis. Plasma concentrations of glucose, total cholesterol, triglycerides (TG), urea, creatinine, albumin, total protein, minerals (Ca, inorganic P, Mg, Na, K, Cl, and Fe), total bilirubin, and some enzymatic activities (amylase, γ-glutamyltransferase, GGT; l-lactate dehydrogenase, LDH; alkaline phosphatase, ALP; l-lactate dehydrogenase, LDH; tartrate-resistant acid-phosphatase, TRAP; alanine aminotransferase, ALT; and aspartate aminotransferase, AST) were measured using an automated analyzer for biochemical chemistry (ILAB Aries, Instrumentation Laboratory, Lexington, MA, USA) working at 37 °C, by colorimetric and enzymatic methods using commercial kits (Instrumentation Laboratory). Plasma globulins were calculated by subtracting albumin from total protein for each blood sample.

Plasma nonesterified fatty acids (NEFA) contents were determined by a commercial kit (NEFA-HR(2), Wako Chemicals GmbH, Neuss, Germany; Richmond, VA, USA), and D-3-Hydroxybutyrate (BHB) using Randox Chemicals kits (RANDOX Laboratories Ltd., Crumlin, UK), adapting them to the clinical chemistry analyzer conditions.

Blood samples without anticoagulant were allowed to separate serum for 6 h after collection, centrifuged at 1850× *g* for 20 min, and frozen at −20 °C until analysis. The serum protein profile was assessed by agarose gel electrophoresis using a standard kit for blood serum proteins (Hydragel 30, Sebia Italia, Firenze, Italy), on an automated multi-parametric agarose gel electrophoresis system (Hydrasis, Sebia Italia). The gels were analyzed using a densitometer and dedicated software (Phoresis, Sebia Italia) to quantify the following fractions: albumin; alpha (α), beta (β), and γ globulins.

The analytical profiles were selected according to our previous studies [33,34] and following the suggestions of Boone et al. [35].

#### 2.1.3. Histological Analysis of Jejunum and Colon

Nine animals, three per treatment, were euthanized by intracardiac lethal injection under total anesthesia according to the current Italian regulations (law number 26/2014, attachment IV). Immediately after sacrifice, a longitudinal incision of the abdominal wall was performed and the whole GIT was removed. Samples of the jejunum and colon were collected from the middle of each considered tract.

Samples were promptly fixed in 10% neutral buffer formalin for 24 h at room temperature and processed for histological purposes. Briefly, they were dehydrated in a graded series of alcohols, cleaned with xylene, and embedded in paraffin.

After dewaxing and rehydration, sections of 5 μm thickness were stained with hematoxylin–eosin (HE) to evaluate intestinal morphology and the general mucosa architecture. Samples were evaluated using a Nikon Eclipse E600 microscope (Nikon, Tokyo, Japan) equipped of a Ds-fi2 camera and the NIS-Elements software package (Nikon). Images were observed at continuous magnifications between 20 and 400.

HE stained sections were used to measure villi height (from the apex of the villus to the villus–crypt junction), crypt depth (from villus–crypt junction to the base of the crypt), and the V:C ratio to evaluate the effects of the diet regimens on the gut health. Values were based on the measurements of 20 villi and 20 crypts per sample.

#### 2.1.4. Microbiological Analysis of Fecal Microbiota

At the end of the testing period of 43 days, samples of fresh feces were collected, directly with anal stimulation, from the control (C) and treated (T2) pigs for microbiological characterization by fluorescence in situ hybridization (FISH) as previously described [36]. The following bacterial groups were searched: Eubacteria (probes EUB338 I, II e III); *Clostridium hystolyticum* (probe Chis150); *Clostridium coccoides–Eubacterium rectale* (probe Erec482); *Faecalibacterium prausnitzii* Cluster (probe Fprau645); *Lactobacillus–Enterococcus* (probe LAB158); *Ruminococcus* (probe Rbro730); *Bacteroide–Prevotella* (probe Bac303); *Bifidobacterium* (probe Bif164); *E. coli* (probe Ecol1513). Briefly, immediately after collection, feces were homogenized and treated in order to detach microbial cells from the fecal particles. Bacterial cells were fixed (PBS 3% paraformaldehyde), and stored (PBS/ethanol 1/1, *w*/*w*) at −20 °C until further processing. Defrosted samples were opportunely diluted, and then bacteria were collected by filtration into polycarbonate filters (0.2 μm pore-size, ISOPORE GTTP02500, Merck Millipore, Darmstadt, Germany). The filters were dehydrated by dipping them in 50%, 80%, and 96% aqueous ethanol. For the in situ hybridization, the filters were covered with hybridization buffer (900 mM NaCl, 20 mM Tris-HCl pH 8.0, 0.01% SDS set to different formamide concentration according to different probes) and labeled probe (50 ng/µL) (MWG-Biotech, Ebersberg, Germany). Hybridization was performed at 46 °C for 4 h. After hybridization, each filter was transferred into a washing buffer (20 mM Tris-HCl pH 8.0, 5 mM EDTA, 0.01% SDS, pH 8.0) with different NaCl concentrations to achieve the appropriate washing stringency, for 10 min at 48 °C. Finally, filters were dried and mounted on a microscope slide with anti-fading oil (Citifluor Ltd., London, UK), and examined with an epifluorescence microscope Axioskope (Zeiss, Oberkochen, Germany) equipped with a 50 W HBO high-pressure mercury lamp (Osram, Munich, Germany) and Zeiss 15 filter set. An eyepiece with a calibrated reticule was used for bacterial counting.

### 2.2. Experiment 2 (Digestibility Trial)

#### Animals, Experimental Design, and Diets

All pigs continued the treatment fed in the post-weaning period. On this assumption, the digestibility trial utilized 18 pigs (6 animals per diet), randomly chosen from the remaining 51 used in the post-weaning trial, in three consecutive digestibility/balances periods. For each digestibility period, two pigs for each treatment were housed individually in metabolic cages for 10 days: 3 days of cage adaptation and 7 days of measurements to determine the digestibility of the diets and the N balance, by total feces and urine collection. The three periods followed one another without interruptions and the first period started when pigs were 101 days old.

For each period, the six pigs involved were weighed at the beginning and at the end of the period.

The three treatments tested were the same used in the post-weaning trial, with diets formulated to fulfill the NRC requirements [32] for growing pigs. The composition of C diet and its chemical analysis is reported in Table 1. The individual daily amounts of each experimental diet were prepared daily; the pigs were fed at a restricted level (daily feed offered 8.6% BW^0.75^ on average) and received two equal meals per day at 08:00 and 18:00 h. Throughout each measurement period, urine was collected individually in a vessel containing 150 mL of a 20% (*v/v*) H_2_SO_4_ solution to maintain a pH below 2.5 and avoid ammonia loss. Urine and feces were weighed daily, sampled (10% and 20% of total weight, respectively, for urine and feces), pooled per pig and frozen (−20 °C) for subsequent chemical analysis. All the diets were sampled daily, pooled, and frozen (−20 °C) for further chemical analysis.

Globally, the average BW of the pigs in the trial was 75 ± 8 kg.

### 2.3. Chemical Analysis

Diets, feed residues, and feces were analyzed for DM content drying at 55 °C in a forced ventilation oven until a constant weight was achieved.

Analytical DM, ash, ether extract (EE), N, and starch contents were determined according the methods proposed by AOAC [37]. Analytical DM was determined by heating at 105 °C for 3 h (method 945.15), ash by incineration at 550 °C for 2 h (method 942.05), EE by solvent extraction (method 920.29), and N (diets, wet fecal samples, and urine) by the Kjeldahl method (method 984.13). Neutral detergent fiber (NDF) and acid detergent fiber (ADF) were analyzed by Ankom^II^ Fibre Analyzer (Ankom Technology Corporation, Fairport, NY, USA) following the procedure of Mertens [38] for NDF and Van Soest et al. [39] for ADF. The gross energy (GE) of feeds, feces, and urine was measured using an adiabatic bomb calorimeter (IKA 4000; Ika, Staufen, Germany).

### 2.4. Statistical Analysis

Data from both trials were analyzed using the GLM procedure of SAS [40]. Least squares mean estimates are reported.

For the post-weaning trial, data were analyzed according to a monofactorial design by using the pen as the experimental unit.

For the digestibility trial, the model initially included the dietary treatment, period, and their interaction. Later, the effects of the period and the interaction were excluded from the model because they were not significant.

The model was:

$$Y_{ij}=\mu +T_{i}+e_{ij}$$

where *Y_ij_* is the dependent variable; *μ* is the general mean; *T_i_* (*i* = 1, 2, 3) is the treatment effect; and *e_ij_* is the residual error.

The statistical analysis of plasma metabolite concentrations and enzyme activities was performed by a linear mixed models using lme4 R package [41], with R Version 3.4.4. (CRAN Garr Mirror, Milan, Italy) according to the model:

Y=μ+TRT+e

where *Y* is the vector of observations of the dependent variable (metabolite or enzyme activity); *μ* is the overall mean; *TRT* is the fixed effect of the dietary supplementation treatment; and *e* is the residual error.

The least square mean (LS mean) and standard error of the mean were calculated using the lsmeans R package. When a significant diet effect was observed, the Tukey test was used to compare means.

For all statistical analyses, significance was declared at *p ≤* 0.05.

## 3. Results

### 3.1. Experiment 1 (Post-Weaning Trial)

#### 3.1.1. Zootechnical Performance

The productive performance of the piglets during the post-weaning trial is reported in Table 2. No differences were recorded for feed intake, final BW, FCR and ADG between treatments during the 43 days of the trial.

#### 3.1.2. Clinical and Biochemical Parameters

The influence of the diet on blood plasma metabolites, minerals and enzyme activities analyzed in the piglets at the end of the post-weaning experiment is summarized in Table 3. Plasma parameters related to energy and lipid metabolism were not significantly affected by diet; however, the glucose level tended to be lower (*p* = 0.078) in the group with the highest dose (T2) of the additive compared to the C and T1 diets.

In regard to plasma nitrogen and protein metabolites, only urea differed significantly between groups, being greater (*p* < 0.05) in T1 piglets compared to T2 ones, and intermediate in the C group. However, both the treated groups showed numerically higher levels of plasma albumin than the control group; although statistical significance was not reached, with a greater number of observations this would have been achieved. Plasma minerals contents were unaffected by supplementation.

The results of the plasma enzyme activities and total bilirubin, as possible biochemical indicators of cellular damage and inflammation, showed no statistically significant differences among the groups, except for the AST and LDH values. Piglets of the T1 group showed the highest activities compared to the C group (AST) and T2 (LDH).

No significant differences were found in the serum protein profile (data not shown), with similar values for alpha (α), beta (β), and γ globulins, indicating substantial comparability between groups in terms of inflammatory conditions and immune stresses.

#### 3.1.3. Histological Analysis of Jejunum and Colon

**Figure 1:**
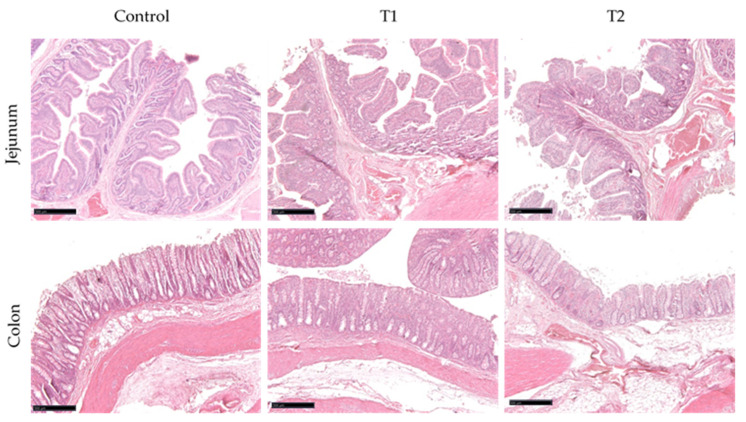
HE-stained sections showing the jejunum and colon morphology of control and treated groups (T1 = Control + 2.5 g GreenFIS®/kg, and T2 = Control + 5 g GreenFIS®/kg) (scale bar 500 μm).

Histological analysis revealed no evident structural changes between the control and treatment groups. No inflammation features were observed, such as villus atrophy, lamina propria widening and epithelial vacuolization (Figure 1). Moreover, villi height, and the villus/crypt ratio remained constant in the jejunum. The V:C ratio was always higher than 3:1, further confirming the animals’ healthy state. The crypt depth also remained unchanged among the different experiments in the colon (Table 4).

#### 3.1.4. Fecal Microbiota Composition

The analysis of microbiological data recorded in pig feces after 42 days of different diets showed a trend towards an increase in bacterial count for all the investigated microbial groups in the pigs fed the T2 diet, compared to the C group; however, the statistical analysis showed significant differences only for the *Ruminococcus* group (Table 5).

### 3.2. Experiment 2 (Digestibility Trial)

The results regarding apparent fecal digestibility are reported in Table 6.

During the digestibility trial, the average pigs’ BW was 74.4, 82.4, and 68.0 kg for C, T1, and T2, respectively. On average, 2.13, 2.25, and 2.08 kg of feed was administered daily for C, T1, and T2, respectively and no leftovers were recorded.

The inclusion of GreenFIS^®^ at both levels increased DM, OM, CP, EE, ash, and GE digestibility compared to C, while no significant differences for these variables were recorded between T1 and T2. Regarding the NDF digestibility, the supplemented diets registered numerically higher values (*p* = 0.059) compared to C. No difference between treatments was detected for ADF digestibility.

The nitrogen balance is reported in Table 7. Regarding the daily nitrogen intake (NI), a significant increase was recorded for T1 compared to C and T2 (*p* < 0.001). Moreover, the retained N (g/d) was higher for T1 compared to C (*p* = 0.031), with T2 being intermediate. However, when the values are expressed in relation to the metabolic weight, thus compensating for the weight of the animals, the NI of T2 was higher than C and T1 (2.46, 2.36, and 2.30 g/BW^0.75^ for T2, T1, and C respectively; *p* = 0.004), and no difference was observed for retained nitrogen. Finally, when the N balance was expressed in relation to the NI, the only difference found was related to the fecal nitrogen: C had a higher excretion of N compared to T1 and T2 (13.0, 9.8, and 10.0 for C, T1, and T2 respectively; *p* = 0.04).

## 4. Discussion

### 4.1. Post-Weaning Trial

The post-weaning trial lasted 43 days, during which the zootechnical performance of piglets was evaluated. The duration of this trial complies with the European Commission Regulation No. 429/2008 [42], which requires long-term studies (minimum 42 days for weaned piglets) for additives affecting animal production or performance.

Zootechnical performance of piglets is an indirect indicator of animal health. In our study, no differences were observed between treatments regarding feed intake, ADG, and FCR. The commercial product tested has been formulated to counteract the health problems related to weaning, especially PWD. During the trial, no signs of PWD were recorded in any of the three groups. The piglets used in the trial came from a farm in excellent sanitary conditions and, during the trial, were housed on an experimental farm where no pigs had been raised for several months. This helped all three groups to have good general health.

Discussing the results of plasma biochemical parameters in piglets fed a blend of active substances instead of a simple comparison between one of them and a control diet is difficult. Therefore, we try to attribute the results on plasma metabolites and enzymes to the action on the complex, with specific reference, where available, to the possible action of a specific component.

The higher plasma urea content observed in the T1 group is not easy to explain. Stensland et al. [43] suggested plasma urea concentration as a marker of a less efficient use of dietary protein or an increase in protein breakdown. Yang et al. [44] reported greater serum urea nitrogen contents in piglets supplemented with chito-oligosaccharides (COS) compared with control piglets, which indicated that COS increased amino acid catabolism in weaned piglets, which was suggested to be due to nitrogen digestibility or catabolism in weaned piglets, which was increased by dietary supplementation with COS. In our trial, if the same explanation could be applied at the T1 results for MOS supplementation, it was not clear why the T2 treatment did not agree with a possible linear effect of this product.

A high level of plasma albumin is certainly positive because its concentration is generally associated with a good absorption of nutrients. In our trial, we found numerically higher values in both treated groups without reaching statistical significance with respect to the C group.

Activities of plasma ALT and AST enzymes in piglets were higher than those recorded by Shu et al. [45]. These serum activities are considered as biomarkers for liver damage. The higher value of AST in T1 than in C was not associated with a similar pattern for ALT; in this way, we cannot derive a specific hepatic problem, also considering the high standard deviation within T1 subjects.

The effects of humic substances on piglet plasma were assessed by Trckova et al. [3]. An anti-inflammatory-like action was reported by their results of decreased serum globulins [3]. In our trial, we observed a decrease (but not significant) in plasma globulins in T1 and T2 compared to the C group, and this fitted the explanation of the mentioned anti-inflammatory effect. We did not find effects of supplementation on plasma minerals, contrary to the results of Trckova et al. [3], who found a positive effect of HS addition on plasma minerals, suggesting a chelating action of HS that led to a higher availability of dietary minerals for intestinal absorption.

Specific effects directly attributable to glyceryl tributyrate on plasma metabolites and enzyme activities were recently reported by Sotira et al. [21]. They reported an increase in plasma glucose in TRIB-treated piglets, but with absolute values lower than ours; we could hypothesize that the lower number of piglets in our trial reduced the possibility of a significant effect between the C group and T1, the group with the highest plasma glucose concentration. Values of plasma urea and ALP were comparable to those reported by Pluske et al. [46]. Plasma AST was higher in the T1 group than in the C group; however, the enzyme activity was quite a bit higher than that reported by Sotira et al. [21]. These values, together with those of plasma LDH activity (higher in T1), but with similar ALT, GGT, and bilirubin across treatments, suggest the absence of a hepatic cellular problem.

In the treatment groups we did not observe inflammation signs [47] nor typical mucosa remodeling as opposed to our previous observations performed in the pig small intestine in the weaning period [48]. However, it is important to note that the mucosa organization was also perfectly healthy in the control group. This suggests that the healthy intestinal state was more likely the result of the clean experimental environment rather than of the diet regimens. Indeed, this is consistent with the drastic reduction of villi height that we observed at the time of weaning in pigs belonging to the same genetic lineage as those used in the current work but were raised in an intensive farm [48]. In those circumstances, we observed a significant reduction in cells expressing *Sox9* and *Hopx* genes, which are intestinal stem and progenitors’ cells, respectively. This suggests that an optimal environment can prevent the damage of the stem cell compartment, possibly caused by a higher load of pathogenic microorganisms.

Determination of bacterial counts by FISH analysis in pig feces after 43 days of different feeding (C vs. T2) showed significant differences only in terms of the quantity of the *Ruminococcus bromii* group, which increased in treated pigs. *Ruminococcus bromii* (Firmicutes, Clostridial cluster IV) shows a superior ability to degrade insoluble starches compared to other gut bacteria [49,50]. It is well known that bacterial fermentation of resistant starch (RS, starch in the diet that escapes digestion by host amylases) in the large intestine leads to the production of volatile SCFA [51]. Resistant starch provides the largest energy source for microbial growth in the colon and its fermentation modulates the activity of the microbiota and provides health benefits to the host [52], resulting in physiological and nutritional effects by microbial metabolites, including the supply of energy from short-chain fatty acids. The role played by *R. bromii* in releasing energy from RS and the decrease in fermentation of RS when this species is low in the intestinal community lead us to consider it as a “keystone” of colon microbiota [53]. The presence of *R. bromii* stimulates the growth of other intestinal bacteria, through the initial degradation of starch, with releases of oligo-saccharides, which act as substrates easily fermented by other bacteria, including large butyrate-producing bacteria [54,55], thus modulating colon microbial metabolism. Therefore, the primary polysaccharide degrader affects the growth and activity of whole intestinal microbiota. This suggests the potentially positive effects of GreenFIS^®^ intake on intestinal microbial ecosystem and so on nutrient digestibility and absorption as found in the subsequently digestibility trial.

### 4.2. Digestibility Trial

All the pigs in this trial were fed the same treatment assigned to them in the first trial. However, contrary to the post-weaning trial, the diet was administered to a restricted level. This was done because the pigs used were bred to be heavy Italian pigs destined for the production of Protected Designation of Origin (PDO) dry-cured hams (Prosciutto di Parma DOP; Prosciutto di San Daniele DOP). The production regulations for these PDO hams provide for specific weights, age of slaughter, and quality of carcasses, criteria that could not be met with an ad libitum diet [56,57,58].

The digestibility trial was conducted in order to evaluate the long-term effects of the commercial blend GreenFIS^®^ on growing pigs, that had already been fed the product in the post-weaning phase. To the best of our knowledge, no studies have assessed blends with similar composition and pigs during the first growing stage. Many studies have investigated the effects of the inclusion of individual components, which are used for the formulation of GreenFIS^®^, and only on piglets in the post-weaning phase as growth promoters and as non-antibiotic feed additives.

Although the effect of a single compound cannot be isolated, the combination of all components (both T1 and T2) determined a higher fecal digestibility of DM, OM, PG, EE, ash, and GE, compared to C. However, the T1 and T2 treatments achieved similar results for the variables listed above.

Regarding the N balance, the higher IN (g/d) was recorded for T1, due to the heavier BW of the pigs, which resulted in a greater feed ingestion, while C and T2 had similar values. This led to a significantly higher value of retained N (g/d) of T1 compared to C, with T2 being intermediate. However, when the retained N is expressed in g/BW^0.75^, only a numerical difference in T1 and T2 treatments compared to C can be observed (*p* = 0.141), probably due to the high individual variability.

## 5. Conclusions

The inclusion of GreenFIS^®^ did not alter the zootechnical performance and the clinical and biochemical parameters of the piglets during the post-weaning phase. From the histological analysis of the jejunum and colon, no differences emerged between treatments; however, T2 increased the microbial population of the *R. bromii* group, considered beneficial for the host’s health, suggesting an effect on pig nutrition of the improvement in the intestinal microbial balance and SCFA production. This could be related to the greater nutrient digestibility of T1 and T2, compared to C, found in the digestibility trial. However, the lack of difference between the two levels of the additive inclusion leads us to assume that the T1 level is sufficient.

The effects of GreenFIS^®^ may have been partially masked by the excellent sanitary conditions of the farm where the piglets came from and of the experimental farm.

## Figures and Tables

**Table 1:** Composition and chemical analysis of the control diets administered in the experiment 1 (post-weaning trial) and in the experiment 2 (digestibility trial).

Item	Experiment 1	Experiment 2
Ingredients (g/kg DM ^1^)		
Corn meal	295	320
Barley meal	156	225
Wheat meal	172	93.0
Wheat bran	74.0	59.0
Beet pulp	29.0	24.5
Soybean hulls	7.0	0
Soybean meal CP ^2^ 48%	49.0	116
Soybean seeds flaked	38.0	9.0
Soybean protein concentrate	38.0	29.0
Oils	29.0	25.3
Milk whey	27.0	32.8
Lactose	16.5	0
Fish meal	14.3	10.0
CaHPO_4_	4.8	6.3
NaCl	4.1	3.5
CaSO_4_	2.9	5.1
Lysine	5.6	4.8
Threonine	2.4	1.8
Methionine	2.1	1.6
Vitamin-mineral mix ^3^	33.3	33.3
Chemical analysis (g/kg DM)		
CP ^2^	183	182
EE ^4^	65.8	58.0
NDF ^5^	124	131
ADF ^6^	50.5	56.3
Ash	54.0	55.8
Lysine	13.9	13.1
Methionine	5.21	4.55
GE ^7^ (MJ/kg DM)	19.04	18.67

^1^ DM: dry matter. ^2^ CP: crude protein. ^3^ Vitamin-mineral mix in experiment 1, provided (per kg): 500 kIU of vitamin A, 60 kIU of vitamin D3, 4000 mg of vitamin E, 150 mg of vitamin K3, 150 mg of vitamin B1, 300 mg of vitamin B2, 2200 mg of niacin, 200 mg of vitamin B6, 5 mg of biotin, 50 mg of folic acid, 2 mg of vitamin B12, 6000 mg of choline chloride, 7000 mg of betaine hydrochloride, 800 mg of calcium pantothenate, 4000 mg of copper sulfate pentahydrate, 3000 mg of zinc oxide, 1500 mg of manganese oxide, 500 mg of ferrous sulfate monohydrate, 10 mg of sodium selenite, 50 mg of calcium iodate anhydrous. Vitamin-mineral mix in experiment 2, provided (per kg): 400 kIU of vitamin A, 48 kIU of vitamin D3, 3200 mg of vitamin E, 120 mg of vitamin K3, 120 mg of vitamin B1, 240 mg of vitamin B2, 1800 mg of niacin, 160 mg of vitamin B6, 4 mg of biotin, 40 mg of folic acid, 1.6 mg of vitamin B12, 4800 mg of choline chloride, 5600 mg of betaine hydrochloride, 540 mg of calcium pantothenate, 3200 mg of copper sulfate pentahydrate, 2400 mg of zinc oxide, 1200 mg of manganese oxide, 400 mg of ferrous sulfate monohydrate, 8 mg of sodium selenite, 40 mg of calcium iodate anhydrous. ^4^ EE: ether extracts. ^5^ NDF: neutral detergent fiber. ^6^ ADF: acid detergent fiber. ^7^ GE: gross energy.

**Table 2:** Productive performance of piglets during the post-weaning trial (43 days).

Item	Diet ^1^			SE	*p*
	C	T1	T2		
Initial BW (kg)	8.9	9.0	9.1	0.286	0.921
Final BW (kg)	39.8	38.7	38.2	1.17	0.678
ADG (kg)	0.717	0.690	0.679	0.025	0.554
Feed intake (kg/d)	4.27	4.13	4.10	0.253	0.776
FCR (kg/kg)	1.492	1.496	1.505	0.022	0.853

^1^ Diet: C = control, T1 = C + 2.5 g GreenFIS^®^/kg, and T2 = C + 5 g GreenFIS^®^/kg.

**Table 3:** Results from the analysis of variance of plasma metabolites and enzymatic activities in piglets at the end of the experimental period.

Item	Diet ^1^			SE	*p*
	C	T1	T2		
Energy and lipid metabolism					
Glucose, mmol/L	8.14	8.75	7.01	0.537	0.078
Total cholesterol, mmol/L	2.88	2.89	2.65	0.114	0.234
Triglycerides, mmol/L	0.755	0.604	0.701	0.170	0.809
NEFA ^2^, mmol/L	0.177	0.176	0.234	0.036	0.428
BHB ^3^, mmol/L	0.073	0.012	0.036	0.032	0.403
Nitrogen and protein metabolism					
Urea, mmol/L	3.92 ^ab^	5.09 ^a^	3.61 ^b^	0.396	0.021
Creatinine, µmol/L	127	150	123	13.2	0.280
Total protein, g/L	65.9	68.0	64.9	1.39	0.270
Albumin, g/L	37.7	40.2	40.1	1.24	0.282
Globulins, g/L	28.2	27.8	24.8	1.71	0.319
Albumin: Globulins	1.42	1.48	1.69	0.116	0.238
Minerals					
Ca, mmol/L	3.10	3.09	2.99	0.058	0.341
P, mmol/L	3.59	3.81	3.46	0.139	0.191
Mg, mmol/L	1.06	1.13	1.09	0.092	0.850
Na, mmol/L	151	151	150	1.33	0.772
K, mmol/L	7.46	8.53	7.72	0.500	0.278
Cl, mmol/L	106	105	106	0.984	0.760
Fe, µmol/L	34.2	35.8	30.9	2.60	0.388
Enzyme activities and bilirubin					
Amylase, U/L	1784	2370	2321	229	0.146
ALT ^4^, U/L	75.9	80.6	80.0	5.02	0.766
AST ^5^, U/L	78.1 ^b^	162 ^a^	96.6 ^ab^	20.2	0.013
GGT ^6^, U/L	27.3	31.4	29.0	3.59	0.714
ALP ^7^, U/L	276	270	256	13.1	0.564
TRAP ^8^, U/L	15.8	28.1	19.2	5.74	0.289
LDH ^9^, U/L	1498 ^ab^	1822 ^a^	1390 ^b^	107	0.017
Total bilirubin, μmol/L	0.410	0.309	0.342	0.113	0.808

^1^ Diet: C = control, T1 = C + 2.5 g GreenFIS^®^/kg, and T2 = C + 5 g GreenFIS^®^/kg. ^2^ NEFA: nonesterified fatty acids. ^3^ BHB: D-3-Hydroxybutyrate (BHB). ^4^ ALT: alanine aminotransferase. ^5^ AST: aspartate aminotransferase. ^6^ GGT: γ-glutamyltransferase. ^7^ ALP: alanine aminotransferase. ^8^ TRAP: tartrate-resistant acid-phosphatase. ^9^ LDH: l-lactate dehydrogenase. ^a,b^ within rows, means without a common superscript differ (*p* < 0.05).

**Table 4:** Evaluation of jejunum and colon histometry among control and treated groups.

Item	Diet ^1^			SE	*p*
	C	T1	T2		
Jejunum					
Villus length (μm)	1288	1257	1214	228	0.935
Crypt depth (μm)	217	195	210	17.4	0.465
Villus/Crypt ratio (V:C)	5.93	6.46	5.78	1.38	0.940
Colon					
Crypt depth (μm)	290	273	303	17.3	0.293

^1^ Diet: C = control, T1 = C + 2.5 g GreenFIS^®^/kg, and T2 = C + 5 g GreenFIS^®^/kg.

**Table 5:** Analysis of the effect of dietary treatment on intestinal microbial composition. Data are given as means per treatment and expressed as (Log_10_ n)/g DM.

Domain/Genus/Species	Diet ^1^		SE	*p*
	C	T2		
*Eubacteria*	10.8	11.0	0.123	0.395
*Bacteroides–Prevotella*	9.07	9.15	0.183	0.833
*Bifidobacterium*	8.71	8.73	0.176	0.837
*Clostridium histolyticum*	8.97	9.06	0.149	0.782
*Clostridium coccoides–Eubacterium rectale*	9.63	9.77	0.269	0.803
*Escherichia coli*	8.31	8.38	0.074	0.632
*Faecalibacterium prausnitzii*	9.48	9.59	0.218	0.821
*Lactobacillus–Enterococcus*	8.77	9.28	0.167	0.100
*Ruminococcus*	8.28	9.09	0.234	0.022

^1^ Diet: C = control, T2 = C + 5 g GreenFIS^®^/kg.

**Table 6:** Apparent fecal digestibility (%) in growing pigs (BW of 74.6 kg, on average) (*n* = 18, six pigs per diet).

Item	Diet ^1^			SE	*p*
	C	T1	T2		
DM ^2^	86.0 ^b^	88.9 ^a^	89.3 ^a^	0.888	0.026
OM ^3^	87.6 ^b^	90.2 ^a^	90.5 ^a^	0.797	0.030
CP ^4^	87.0 ^b^	90.2 ^a^	90.0 ^a^	0.939	0.040
EE ^5^	82.7 ^b^	87.3 ^a^	85.8 ^a^	1.05	0.017
NDF ^6^	45.7	53.6	57.7	3.59	0.059
ADF ^7^	37.2	38.3	43.2	4.20	0.124
Ash	58.1 ^b^	67.6 ^a^	68.6 ^a^	2.70	0.019
GE ^8^	86.2 ^b^	89.1 ^a^	89.5 ^a^	0.873	0.024

^1^ Diet: C = control, T1 = C + 2.5 g GreenFIS^®^/kg, and T2 = C + 5 g GreenFIS^®^/kg. ^2^ DM: dry matter. ^3^ OM: organic matter. ^4^ CP: crude protein. ^5^ EE: ether extracts. ^6^ NDF: neutral detergent fiber. ^7^ ADF: acid detergent fiber. ^8^ GE: gross energy. ^a,b^ within rows, means without a common superscript differ (*p* < 0.05).

**Table 7:** Nitrogen balance in growing pigs (74.6 kg, on average) (*n* = 18, six pigs per diets).

Item	Diet ^1^			SE	*p*
	C	T1	T2		
N balance, g/d					
Intake (NI)	57.9 ^b^	64.8 ^a^	58.2 ^b^	0.979	<0.001
Fecal	7.53	6.37	5.81	0.558	0.085
Urinary	28.9	30.1	27.0	1.79	0.436
Retained	21.5 ^b^	28.3 ^a^	25.4 ^ab^	1.70	0.031
N balance, g/BW^0.75^					
Intake	2.30 ^b^	2.36 ^b^	2.46 ^a^	0.031	0.004
Fecal	0.297	0.232	0.247	0.021	0.095
Urinary	1.14	1.10	1.14	0.056	0.820
Retained	0.864	1.03	1.07	0.081	0.141
N balance, % NI					
Fecal	13.0 ^a^	9.83 ^b^	10.0 ^b^	0.939	0.040
Urinary	49.7	46.4	46.4	2.62	0.559
Retained	37.3	43.7	43.6	2.77	0.221

^1^ Diet: C = control, T1 = C + 2.5 g GreenFIS^®^/kg, and T2 = C + 5 g GreenFIS^®^/kg. ^a,b^ within rows, means without a common superscript differ (*p* < 0.05).

## Data Availability

Not applicable.
